# Efficacy of Magnesium Sulfate on Maternal Mortality in Eclampsia

**DOI:** 10.7759/cureus.17322

**Published:** 2021-08-20

**Authors:** Jaskamal Padda, Khizer Khalid, Lanson B Colaco, Sandeep Padda, Nymisha L Boddeti, Armughan S Khan, Ayden Charlene Cooper, Gutteridge Jean-Charles

**Affiliations:** 1 Internal Medicine, JC Medical Center, Orlando, USA; 2 Internal Medicine, Advent Health & Orlando Health Hospital, Orlando, USA

**Keywords:** magnesium sulfate, mgso4, eclampsia, preeclampsia, cost-effectiveness, maternal mortality, obstetric medicine, risk of seizure

## Abstract

Eclampsia is a common complication of preeclampsia patients and can be life-threatening for both the mother and the fetus. Hence, timely intervention and appropriate management of this detrimental condition are extremely crucial. Eclampsia is described as the occurrence of generalized convulsions in patients with preeclampsia. Magnesium sulfate (MgSO_4_) is the drug of choice for treating and preventing eclampsia. This review aims to study and analyze the available literature on the pathogenesis of eclampsia, the pharmacology of MgSO_4_, and its effectiveness in the management of eclampsia. Other proposed treatments and their comparative study with MgSO_4_ are also discussed. Additionally, we examine the data regarding the impact of eclampsia, its public health burden, and the cost-effectiveness of MgSO_4_. One of the major drawbacks associated with the use of MgSO_4_ in low-income countries has been the cost of treatment and the lack of resources. We have analyzed the trials that have proposed alternate treatment regimens which could shape new guidelines to resolve these issues. For this review, we extensively studied abstract and full-text articles from multiple databases. This article discusses the pathophysiology of eclampsia, the pharmacology of MgSO_4_, the issues surrounding eclampsia management, and how MgSO_4_ benefits these patients.

## Introduction and background

Eclampsia is a known deleterious sequela of preeclampsia. It has been defined as new-onset generalized tonic-clonic seizures in patients with preeclampsia. Preeclampsia is the occurrence of hypertension (HTN) after 20 weeks of gestation with concurrent proteinuria/end-organ dysfunction [1]. Annually, both eclampsia and preeclampsia account for nearly 63,000 maternal deaths worldwide [2]. A study conducted by the Centers for Disease Control and Prevention (CDC) found an overall case-fatality rate of preeclampsia and eclampsia to be 6.4 per 10,000 cases at delivery. This study also found an increased risk of death in patients at 20-28 weeks of gestation and 3.1 times increased incidence of preeclampsia/eclampsia in black women compared to white women [3].

Eclamptic seizures are a medical emergency and can arise after 20 weeks of gestation, either antepartum, intrapartum, or postpartum. They require urgent intervention to prevent death in the mother and fetus [1]. Magnesium sulfate (MgSO_4_) has been proven to be an effective first-line treatment for the prevention and treatment of eclampsia [1,4]. It should be continued for at least 24 hours from the time of the last seizure and the patient should be monitored closely for toxicity [1]. Even with the risk of side effects and toxicity, many studies have proven that MgSO_4_ is much more superior in the treatment of eclampsia compared to other drugs [4,5]. Nevertheless, the prevalence of eclampsia has decreased because of enhanced prenatal care, judicious use of medical therapy (blood pressure control, seizure prophylaxis, etc.), and conducting term deliveries by either induction or cesarean section [6]. In this review, we discuss the mechanism of eclampsia, the pharmacokinetics and pharmacodynamics of MgSO_4_, the efficacy of MgSO_4_ in reducing mortality in eclamptic patients, and the challenges encountered in MgSO_4_ administration.

## Review

Pathophysiology of eclampsia

Although a comprehensive review of the pathophysiology is beyond the scope of this article, a brief mention of some of the key elements involved in the pathophysiology of eclampsia has been discussed. Eclampsia is a complication of preeclampsia, which is why it is important to understand the pathogenesis of preeclampsia. The placenta is considered to play a significant role in the pathophysiology of preeclampsia. After the placenta is delivered, the symptoms subside quickly [7]. The primary issue is that placental-derived factors released into the maternal circulation cause diffuse endothelial dysfunction, vasoconstriction, and increased vascular permeability, resulting in HTN, proteinuria, and edema [8].

**Figure 1:**
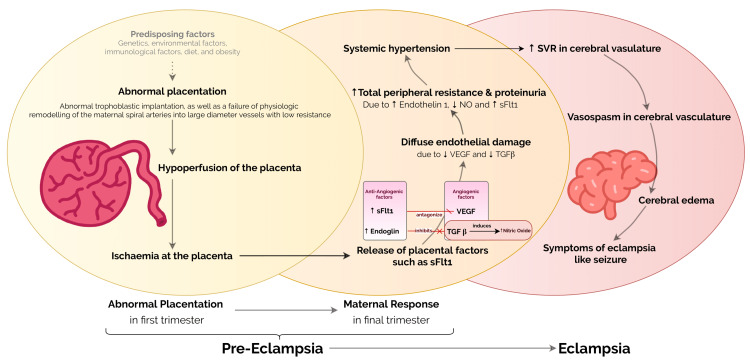
Pathophysiology of preeclampsia and eclampsia. VEGF: vascular endothelial growth factor; NO: nitric oxide; sFltl: soluble FMS-like tyrosine kinase; TGFβ: transforming growth factor-beta; SVR: systemic vascular resistance The image was created by the author (Lanson B. Colaco, MBBS).

The main pathophysiological occurrences can be understood in a simplified manner using the widely accepted “two-stage theory,” which states that preeclampsia develops in two stages. The first is decreased placental perfusion (Stage 1), which results in the release of factors that cause generalized systemic pathology (Stage 2) [9]. The rest of the pathophysiology is explained in Figure 1 [8-11].

According to studies, eclampsia is caused by both cerebral edema and vasospasm. Edema can be generalized or localized, such as in the occipital lobes, white matter, or watershed regions. Vasospasm has been observed in both angiographic and Doppler studies leading to ischemia, causing reduced perfusion of the brain. All of this culminates in edema and microinfarcts (and, on rare occasions, hemorrhage) in these patients [12-15]. Because cerebral edema develops within the restricted area of the skull, it causes progressive brain compression as well as the usual neurologic symptoms of eclampsia such as headache, nausea, vomiting, cortical blindness, and convulsions [16,17].

Pharmacokinetics and pharmacodynamics of magnesium sulfate

Regimens

MgSO_4_ is usually administered by either intramuscular (IM) or intravenous (IV) routes. The two standard regimens that are most commonly used for the management of severe preeclampsia and eclampsia are predominantly the IM Pritchard regimen and the exclusively IV Zuspan regimen [18]. The Pritchard regimen involves administering two loading doses of MgSO_4_, consisting of a slow IV dose of 4 g over five to ten minutes, immediately followed by an IM dose of 10 g divided into 5 g in each buttock. This is then followed by maintenance dosing with 5 g IM into the alternate buttocks every four hours [19]. In the Zuspan regimen, a single loading dose of 4 g is administered as a slow IV infusion over five to ten minutes, followed by an hourly maintenance infusion of 1-2 g by a controlled infusion pump [20]. The serum concentrations of magnesium obtained from the Pritchard regimen fluctuate more often compared to the Zuspan regimen [21].

Pharmacokinetics

After administration, about 40% of plasma magnesium becomes protein-bound, while the unbound ionized magnesium increases proportionately to the total serum concentration of magnesium [22]. The free magnesium ions diffuse into the extravascular space, into the bone, and across the placenta and fetal membranes to enter the fetus and amniotic fluid [23-25]. Magnesium is eliminated almost exclusively by the maternal kidneys, with almost 90% of the dose excreted in the urine during the first 24 hours after an IV infusion [26]. A plasma concentration of 1.8-3.0 mmol/L is recommended for the treatment of eclamptic seizures while carefully monitoring for toxicity beyond this recommended concentration. The first warning of imminent toxicity in the mother is the loss of the patellar reflex at concentrations between 3.5 and 5 mmol/L [26]. Respiratory paralysis and cardiac arrest may occur at supratherapeutic concentrations beyond 5 mmol/L [27]. Therefore, close monitoring for the loss of deep tendon reflexes, respiratory rate <12 breaths/minute, urine output <30 mL/hour, and high plasma concentrations are of paramount importance [28].

Pharmacodynamics

The anticonvulsant mechanism of MgSO_4_ is attributed to its actions upon the central nervous system (CNS) and vascular endothelium, while its effects are mediated through the neuromuscular junction (NMJ). Generalized CNS depression occurs via voltage-dependent N-methyl-D-aspartate (NMDA) receptor blockade [29] and NMJ blockade by decreasing calcium conductance, acetylcholine release, and motor endplate excitability to acetylcholine release [30]. It is suggested to cause vasodilation by stimulating prostacyclin I2 and nitric oxide synthesis in vascular endothelial cells [31]. In addition, it is understood that this vasodilatory effect of MgSO_4_ upon the smaller-diameter intracranial vessels functions to reduce cerebral ischemia when used in the prophylaxis and treatment of eclampsia [32].

Efficacy of magnesium sulfate in eclampsia

MgSO_4_ has been proven to be an effective first-line treatment for the prevention and treatment of eclampsia. Nonetheless, studies are still being conducted to gauge the degree of effectiveness in comparison to other anticonvulsant drugs. The Cochrane Library was searched for trials that prove the effectiveness of MgSO_4_. The results show that compared to the placebo/no anticonvulsant group, the mortality rate was reduced by 46%, the risk of eclampsia was reduced by half, and there was decreased risk of placental abruption. The risk of eclampsia was also found to be decreased in patients receiving MgSO_4_ in comparison to anticonvulsant therapy [4].

Lucas et al. compared the efficacy between MgSO_4_ and phenytoin. Results showed that 10 out of 1,089 women who were placed in the phenytoin group had eclamptic convulsions, whereas zero of 1,049 women placed in the MgSO_4_ group had any eclamptic activity. These results corroborate the long-practiced use of MgSO_4_ in the prevention of eclampsia [5].

A retrospective study conducted in Nigeria by Okereke et al. from 2008 to 2009 focused on the reduction of maternal mortality in eclamptic patients with the use of MgSO_4_. The study compared the effectiveness of MgSO_4_ with diazepam. Participants included 1,233 eclampsia patients in the diazepam group and 996 eclampsia patients and 47 preeclampsia patients in the MgSO_4_ group. Results showed a drastic decrease in the eclampsia case-fatality rate from 20.9% to 2.3% among patients in the MgSO_4_ intervention group [33]. MgSO_4_ has been recognized as the drug of choice for the treatment of eclampsia and preeclampsia by the World Health Organization (WHO) [34]. Systematic reviews and randomized controlled trials have proven the effectiveness of MgSO_4_ in preventing these conditions [35]. The evidence, therefore, verifies that MgSO_4_ is effective in decreasing maternal mortality in eclampsia.

Prophylactic usage of magnesium sulfate

MgSO_4_ is used for the prevention of eclampsia in high-risk patients. Despite the drug’s proven efficacy, there has been an ongoing debate regarding the correct dosage regimens and their feasibility in resource-limited areas [36]. In this section, we will review the studies that have delved into this topic.

Initially, some physicians continued to use antiepileptics to prevent the occurrence of seizures in high-risk patients. However, this use was not backed by evidence. A comparative study was done by Lucas et al. in 1995 which compared phenytoin and MgSO_4_ for the prevention of eclampsia in hypertensive patients. They divided pregnant hypertensive women into two groups, one group was allocated treatment with phenytoin (n = 1,089) and the other with MgSO_4_ (n = 1,089). A total of 10 patients from the phenytoin group developed convulsions whereas none of the MgSO_4_ patients developed any symptoms indicative of eclampsia [5]. This study was followed by the MAGPIE trial which selected pregnant patients with HTN from 33 countries. In total, 5,071 patients were allocated to MgSO_4_, while 5,070 patients were given placebo treatment. The study proved that the use of MgSO_4_ reduces the risk of developing eclampsia by 50% and lowers the maternal mortality rate. Since this landmark trial, the WHO has promoted the use of MgSO_4_ for seizure prophylaxis [37].

The traditional regimens of MgSO_4_, such as those described by Pritchard and Zuspan, require the availability of experienced personnel for administering the medication and monitoring drug toxicity. In low-income areas with a scarcity of resources, the attainability of such ideal conditions can be difficult [38]. This has led to physicians studying and comparing low-dosage regimens with the established regimens for the treatment of eclampsia.

The Dhaka regimen utilizes a 10 mg loading dose of MgSO_4_, followed by 2.5 mg given four hours. This is approximately half the dose of the Pritchard regimen and has been found to be equally effective in reducing recurrent seizures [39]. Many studies have now shown that low-dose MgSO_4_ regimens such as Dhaka regimen, Sokoto regimen, low-dose loading, and maintenance regimens can also adequately manage eclampsia [38]. Such regimens reduce the strain on healthcare resources while maintaining treatment quality and a low incidence of drug toxicity events.

Most of these comparative studies have been carried out in developing countries of Asia. Begum et al. compared a lower-dosage regimen with the higher-dosage regimens and showed that there was equal efficacy in treating eclampsia with both regimens [40]. More recently, a randomized control trial conducted in Nepal compared a single loading dose of MgSO_4_ with the standard regimen. The trial demonstrated that a single dose is as effective as standard treatment in preventing eclampsia and can be alternatively used for prophylaxis. The results of this trial backed similar findings of Shoaib et al. (Pakistan), Rangana et al. (India), and Murthy et al. (India) reported previously [36,41].

With the above findings in mind, the PIPES trial in 2018 went a step further to compare the single-dose regimen with the low-dose Dhaka regimen in a randomized control trial. Although results did not yield statistically significant differences between the two groups, it was deduced that the shorter regimen of MgSO_4_ is a viable replacement for the low-dose Dhaka regimen [38]. The aforementioned data demonstrate the efficacy of single, low-dose MgSO_4_ for the prevention of eclampsia. Larger polycentric trials may be needed to compare it with the 24-hour standard regimen to gain more clarity in updating treatment guidelines.

Cost-effectiveness

The WHO has acknowledged MgSO_4_ as the safest, most efficient, and cost-effective medication for the treatment of preeclampsia and eclampsia and has placed it on the Essential Medicines List (EML) for this specific use. Although it is the standard treatment in developed countries, other medications such as diazepam and phenytoin are still widely used in underdeveloped countries. MgSO_4_ approximately costs $0.10/mL. From 1995 to 2002, sizable clinical trials found MgSO_4_ to be most effective in the treatment of preeclampsia and eclampsia compared to other treatment modalities. These trials exhibited more than a 50% reduction in the incidence of eclampsia and a 46% reduction in maternal mortality [42].

While MgSO_4_ is the drug of choice in the treatment of eclampsia, cost remains an issue in developing countries. Routine checking of serum magnesium levels can also become costly. Simon et al. of the MAGPIE trial stated that MgSO_4_ is most cost-effective when it is limited to patients with severe preeclampsia, which is the precursor to eclampsia. This trial stated that to prevent one case of eclampsia, the cost in USD for 2001 was $21,202 in high, $2,473 in middle, and $456 in low-income countries. The cost-effectiveness of MgSO_4_ would be improved if used only in cases with severe preeclampsia [4].

Another study based on the MAGPIE trial collected data from 9,996 pregnancies: 1,195 from high gross national income (GNI), 5,571 from middle GNI, and 3,230 from low GNI countries. This study assessed the cost of treatment for eclampsia in patients from countries categorized into three distinct groups based on their national income. The results indicated that the number needed to treat in order to prevent one case of eclampsia is 324 in high GNI, 184 in middle GNI, and 43 in low GNI countries. The general cost to counteract one case of eclampsia was $21,202 in high, $2,473 in middle, and $456 in low GNI countries. If it is decided to focus MgSO_4_ treatment on severe preeclampsia, these costs would be lowered to about $12,942, $1,179, and $263, respectively. If MgSO_4_ prophylaxis was administered to women with severe preeclampsia, the cost per case would decrease approximately by half. The average cost of MgSO_4_ treatment which includes the drug and administration is anticipated as $86 for high GNI, $17 for middle GNI, and $13 per person for low GNI countries. It is known that the majority of the costs associated with MgSO_4_ treatment include drug administration instead of the drug itself, that is, 77%, 82%, and 62%, respectively, in high GNI, middle GNI, and low GNI countries [43].

Because drug administration carries most of the weight in terms of cost, many studies have focused on finding alternative methods of drug delivery. If infusion pumps are not readily available for the administration of MgSO_4_, physicians would have to revert to IV delivery methods, or painful, large-volume IM injections. Another method is a syringe pump known as AutoSyp. This invention was designed to be low-cost, accurate, and low-powered to bypass the cost restrictions in countries with low GNI [44]. Therefore, it has been established that the drug MgSO4 itself is cost-effective, but the means of administering it is expensive. MgSO_4_ can be made more cost-effective if given prophylactically to preeclamptic patients and if a more affordable method of administration can be recognized.

Anticonvulsants in the treatment of eclampsia

MgSO_4_ is the drug of choice to treat patients with eclampsia. It has been proven to be a better agent than other anticonvulsant medications such as benzodiazepines and phenytoin. While it was initially believed that eclamptic seizures have similar pathophysiology as other convulsive diseases, the usual antiseizure medications have not shown to be as beneficial as MgSO_4_ in controlling eclamptic seizures suggesting otherwise [4,5,45].

MgSO_4_ initially came to the scene in 1906 after a successful trial was published. It gained prominence in the United States and the United Kingdom during the 1920s, but since the arrival of diazepam and later phenytoin, there persisted a hesitance in the use of MgSO_4_ for treating eclamptic seizures. This reluctance stemmed from the widespread use of these medications for other forms of seizures, their cost-effectiveness, and availability [45]. The controversy largely ended after a landmark randomized trial was conducted in 1995 (Collab trial) which demonstrated MgSO_4_ to be superior to diazepam and phenytoin [45]. Trials that followed solidified this evidence. In this section, we will be discussing the results of these trials.

Magnesium Sulfate Versus Diazepam

Diazepam is a cost-effective and readily available agent, especially in rural obstetric centers. It can control convulsions during eclampsia and can treat HTN and tachycardia [46]. Compared to MgSO_4_, diazepam does not sufficiently reduce recurrent seizures during an eclamptic episode. A 52% risk reduction (95% confidence interval [CI] = 64-37% reduction) was reported with the use of MgSO_4_ [45]. Furthermore, patients treated with diazepam usually required additional therapy to adequately control seizures. MgSO_4_ has also been shown to have a significantly lower mortality rate compared with diazepam (relative risk [RR] = 0.59; 95% CI = 0.37-0.94) [47]. Diazepam has been associated with depressive effects on infants. Infants of mothers who had been treated with diazepam had APGAR scores less than seven at one minute and longer stay in neonatal care units. These low scores persist at five minutes as well [45,47].

Magnesium Sulfate Versus Phenytoin

MgSO_4_ has proven to be considerably superior to phenytoin in terms of reducing the incidence of recurrent seizures (RR = 0.31; 95% CI = 0.20-0.47). In addition, those treated with phenytoin have been seen to require auxiliary medications to aid the control of convulsions [45,47]. Improved maternal morbidity outcomes have been observed with the use of MgSO_4_. Those designated to MgSO_4_ had a significant reduction in their risk of developing pneumonia (RR = 0.44; 95% CI = 0.24-0.79), requiring intubation (RR = 0.66; 95% CI = 0.49-0.90), and intensive care unit admission (RR = 0.67, 95% CI = 0.50-0.89). Although no significant changes in maternal mortality are observed when comparing the two medications [4,45,47]. Phenytoin has substantial effects on infant morbidity as well. Fewer infants of mothers treated with MgSO_4_ had APGAR scores less than seven at one minute, required intubation at the time of birth, or required admission in neonatal special care nursery [45,47].

Data table

Table 1 lists peer-reviewed articles published in English and relevant to the topic. A thorough search on databases such as PubMed, PubMed Central, and Google Scholar was conducted. The following inclusion criteria were used and then decided upon based on the authors’ discretion: women without eclampsia; women with preeclampsia; primary objectives including the development of eclampsia, other life-threatening complications, or death; treatment group receiving MgSO_4_ as per the local regimen; and a sample size of >100.

**Table 1:** Summary of included studies involving the usage of magnesium sulfate in preeclampsia to prevent or treat eclampsia. PE: preeclampsia; MgSO_4_: magnesium sulfate; IV: intravenous; IM: intramuscular; HELLP: hemolysis, elevated liver enzymes, low platelet count syndrome

Study	Sample size	Study participants	Intervention: Group A	Intervention: Group B	Primary outcomes in women	Objective	Conclusion
MAGPIE Trial Duley et al. (2002) [36]	10,141	PE with uncertainty on whether to use MgSO_4_	MgSO_4_: 4 g IV bolus. Subsequently, either 1 g/hour IV infusion or 10 g (or 5 g) IM bolus, followed by 5 g every 4 hours. Continued for 24 hours	Control: Placebo with a matching regimen	Eclampsia and death	To evaluate the effects of MgSO_4_ on women and their babies irrespective of whether treatment was started before or after delivery and irrespective of any previous anticonvulsant therapy	MgSO_4_ decreases the risk of eclampsia by half, and probably reduces the risk of maternal death
Lucas et al. (1995) [5]	2,138	BP ≥140/90 mmHg	MgSO_4_: 10 g (50% solution) IM (5 g in each buttock), followed by 5 g IM every 4 hours. In case of severe PE, an additional 4 g IV (20% solution) before the first IM dose	Phenytoin: 1,000 mg IV over 1 hour. After 10 hours, 500 mg orally	Eclampsia	To compare MgSO_4_ with phenytoin in preventing seizures in hypertensive women during labor	MgSO_4_ is superior to phenytoin for prophylaxis against eclampsia
Nimodipine Study Group Belfort et al. (2003) [48]	1,650	PE: planned delivery and no previous MgSO_4_	MgSO_4_: Either 4 g IV followed by 1 g/hour, or 6 g IV followed by 2 g/hour	Nimodipine: 60 mg every 4 hours orally	Eclampsia, stroke, coagulopathy, respiratory problems, and cardiac failure	To determine whether nimodipine is more effective than MgSO_4_ for seizure prophylaxis in women with severe PE	MgSO_4_ is a superior prophylactic medication to nimodipine against seizures in women with severe PE
Coetzee et al. (1998) [49]	699	Severe PE: at least 2 of DBP ≥110 mmHg, significant proteinuria, symptoms of imminent eclampsia	MgSO_4_: 4 g IV in 200 mL saline over 20 minutes, followed by 1 g/hour (200 mL over 4 hours) until 24 hours after delivery	Control: Placebo with a matching regimen	Death and eclampsia	To see if prophylactic IV MgSO_4_ administration reduces the incidence of eclampsia in women with severe PE	IV MgSO_4_ treatment significantly reduced the development of eclampsia in women with severe PE
PIPES Trial Keepanasseril et al. (2017) [37]	402	Singleton pregnancy complicated by severe PE	MgSO_4_: Low-dose Dhaka regimen	MgSO_4_: Only loading-dose regimen	Eclampsia	To study the efficacy of the loading-dose regimen versus the low-dose Dhaka regime in seizure prophylaxis in women with severe PE	The less resource-intensive and shorter regimen of MgSO_4_ using only the loading dose in the prevention of seizure in PE is an effective alternative to the commonly used low-dose Dhaka regimen
Livingston et al. (2001) [50]	222	Mild PE during labor	MgSO_4_: 6 g IV, followed by infusion of 2 g/hour	Control: Placebo with a matching regimen	Eclampsia and HELLP	To see if MgSO_4_ prevents disease progression in women with mild PE	MgSO_4_ did not demonstrate a major impact on disease progression in women with mild PE

Clinical impact

According to a systematic review by Behran et al., a significant number of women with mild preeclampsia show no warning symptoms of developing eclampsia. Because there is a relatively low risk of MgSO_4_ toxicity, it is recommended to give MgSO_4_ prophylaxis to all women with mild preeclampsia [51]. MgSO_4_ reduces the incidence of eclampsia and lowers maternal mortality, and therefore should be considered for women with preeclampsia who are at risk of developing eclampsia. Its low cost, easy availability, and relatively simple procedure for medication administration and monitoring are advantages. This procedure can be administered by medical personnel, nursing, or even midwifery staff if properly trained [37].

## Conclusions

Eclampsia is a very serious complication of preeclampsia that presents after 20 weeks of gestation. It is associated with increased seizure activity, HTN, and end-organ damage. The CDC has reported a case-fatality rate of 6.4 per 10,000 cases of preeclampsia and eclampsia at delivery. The drug of choice to conquer these effects is MgSO_4_ which exerts its effects on the CNS through the blockade of the NMDA receptors. The Cochrane Library analyzed many trials that proved the efficacy of MgSO_4_ in the treatment of maternal eclampsia. They proved that the mortality rate decreased by 46% and the risk of eclampsia was reduced by half in patients receiving treatment with MgSO_4_. Many other studies compared the effectiveness of MgSO_4_ with other anticonvulsant medications and the results consistently show MgSO_4_ as being the most efficacious agent. Prophylactic usage has also been widely recommended by many studies, such as the MAGPIE trial, which suggests that if MgSO_4_ is given to all mildly preeclamptic patients, the risk of progressing to severe preeclampsia and eclampsia will decrease substantially.

Unfortunately, the use of MgSO_4_ is constrained in developing countries by the high costs associated with the infusion pump required to administer the drug as well as the intensive monitoring for signs of toxicity. Given that preeclampsia/eclampsia remains one of the most common causes of maternal mortality in low- and middle-income countries, solutions have been proposed to improve the cost-effectiveness of MgSO_4_, such as limiting its use to severe preeclampsia/eclampsia along with using the IM route and employing alternative low-cost methods of drug delivery. Ultimately, it is well established that MgSO_4_ is an excellent drug in reducing maternal mortality in eclampsia, concluding that MgSO_4_ is an effective treatment for the prevention and treatment of eclampsia. With ongoing debate regarding the correct dosage regimens and their feasibility in resource-limited areas, further investigation is recommended for the use of low-dose prophylactic use of MgSO_4_ in eclampsia.
